# Ultrasound-guided percutaneous biopsy for focal liver lesions: Adverse events and diagnostic yield in a single-centre analysis

**DOI:** 10.1371/journal.pone.0304026

**Published:** 2024-05-22

**Authors:** Theresa Pöschel, Valentin Blank, Tobias Schlosser, Thomas Lingscheidt, Albrecht Böhlig, Johannes Wiegand, Thomas Karlas

**Affiliations:** 1 Division of Gastroenterology, Department of Medicine II, Leipzig University Medical Centre, Leipzig, Germany; 2 Division of Interdisciplinary Ultrasound, Department of Internal Medicine I (Gastroenterology, Pneumology), University Hospital Halle, Halle (Saale), Germany; 3 Institute of Pathology, Leipzig University Medical Centre, Leipzig, Germany; 4 Division of Hepatology, Department of Medicine II, Leipzig University Medical Centre, Leipzig, Germany; 5 Department of Internal Medicine, Community Hospital Delitzsch, Delitzsch, Germany; Al-Azhar University, EGYPT

## Abstract

**Purpose:**

Ultrasound-guided biopsy of focal liver lesions (FLL) is a well-established procedure with crucial impact on therapeutic decisions. The safety and accuracy depend on needle type, tumour location and comorbidities. Modern oncological concepts often require large tumour specimens which may increase the procedural risk.

**Materials and methods:**

We retrospectively collected data from consecutively scheduled ultrasound-guided FLL biopsies performed in an interdisciplinary ultrasound unit at a university hospital from 2015–2020. We analysed complication rates, diagnostic accuracy, and patient outcome in a one-year period.

**Results:**

Of 426 scheduled interventions, 339 were included: 322 primary biopsies (40% female, median age 65 years, median BMI 25.4 kg/m^2^) and 17 rebiopsies in cases with undetermined diagnosis. Indications comprised 309 (96%) cases with suspected malignant lesions. Important comorbidities were type 2 diabetes (n = 107, 33%) and cirrhosis (n = 64, 20%). A conclusive histopathological diagnosis was achieved in 270 (84%) cases with a weak association with lesion size (OR 1.12 per cm, 95%CI 0.99–1.27). Greater BMI (OR 0.60 per 10 BMI points, 95%CI 0.34–1.05) showed a trend towards an insufficient diagnosis. Relevant complications occurred in 8 (2.5%) cases (2 major; 1 life-threatening). Multiple passes showed a trend towards adverse events (OR 2.32 for > 1 pass, 95%CI 0.99–5.42). 93 (29%) patients died during a median follow-up of 171 days.

**Conclusion:**

Ultrasound-guided FLL biopsy is an efficient and safe diagnostic measure. The limitations of the procedure and its associated risks should be considered in patients with advanced malignancies.

## Introduction

Ultrasound is essential for the differential diagnosis of focal liver lesions (FLL) [1]. If conventional and contrast-enhanced ultrasound (CEUS) imaging do not lead to a definite diagnosis, a histological specimen may be necessary. Among the various approaches for collecting material, percutaneous ultrasound-guided biopsies of FLL play a key role in the clinical routine [2]. Therefore, technical and clinical guidelines have been established [2, 3]. Full core needle biopsy using an 18-gauge needle has emerged as the diagnostic standard approach for percutaneous collection of specimens from FLL [4]. In Germany, ultrasound-guided interventional procedures of the abdomen are frequently performed by specialists in internal medicine, surgeons and radiologists [5, 6].

Although ultrasound-based diagnostic procedures are widely accepted as a first-line approach, safety, diagnostic accuracy and patient comfort may vary considerably among different cohorts and biopsy indications [7–9]. Previous studies that analysed the performance of ultrasound-guided core biopsy for FLL revealed low complication rates and a moderate rate of indication for repetitive biopsies [10–15]. However, the majority of these data were collected before the advance of modern molecular pathology and individualized target-tailored oncologic therapies, that often require large and multiple tumour specimens [16, 17]. Such therapeutic options have pushed the frontline for complex systemic interventions before best supportive care concepts are implemented. Thus, biopsies of FLL are nowadays more frequently attempted in patients with advanced stages of their disease and a considerable burden of comorbidities [18–20]. This extension of biopsy-indication requires a re-assessment of ultrasound-based biopsy safety and diagnostic performance in the era of modern oncology. Therefore, we conducted a retrospective analysis of ultrasound-guided biopsies of FLL at our tertiary medical centre.

## Materials and methods

### Ethics, data protection and registration

The study protocol was approved by the institutional review board (IRB) of the University of Leipzig (ethics committee vote 113/21-ek). Due to the retrospective nature of the study, the need for informed consent was waived by the IRB.

The original data were accessed from 01/05/2021 to 31/05/2022. Only TK and TP had access to the original patient files. The data were pseudonymized immediately after collection and the further analysis was performed anonymously.

The study was registered in the German Register of Clinical Studies (DRKS): DRKS00024936.

### Case identification

We reviewed patient data from the clinical information system i.s.h.med (SAP, Walldorf, Germany) and ViewPoint Vs. 5 (GE Healthcare, Solingen, Germany). We identified all patients (age ≥ 18 years) scheduled for ultrasound-guided biopsy of FLL at the interdisciplinary ultrasound unit in the period from 01/01/2015 to 31/12/2020. We did not include patients with biopsy of liver parenchyma only nor biopsies of other organs.

Data obtained from electronic medical records included sex, age, BMI, medical history (coronary heart disease, chronic kidney disease [21], type 2 diabetes, previous diagnosis of malignant disease), anti-coagulation medication, indication for FLL biopsy and recent laboratory tests (performed ≤7 days before biopsy; in single cases (n = 9) with stable condition an interval ≤14 days was accepted).

### Procedural data

Procedural data included preinterventional substitution of haemostatics agents, characteristics of the target lesion (solitary or multiple, size, localisation (hepatic segment following the Couinaud classification [22]), and potential high-risk constellations like proximity to blood vessels or capsule).

All FLL biopsies were performed with ultrasound guidance according to standard procedure recommendations [2] using 16/18/20-gauge needles, either core biopsy or other techniques as selected by the operator. The following data was collected: needle type and diameter including the predefined cylindrical sample length, number of passes and specimens. The experience of the operator was classified according to the number of performed biopsies during the analysed period.

### Complications and outcome

All patients underwent a control ultrasound examination 18–24 hours after intervention or earlier in case of suspected adverse event (AE), comprising the assessment of active bleeding, postinterventional haematoma, or free peritoneal fluid. Biopsy complications were also recorded based on entries in the patient chart or diagnosis at discharge, especially the need for transfusion or analgetic treatment. We classified AE into five categories: no complication, minor complication (treatable on an outpatient basis), major complication (need of longer hospitalisation), life-threatening complications with need for intensive care or interventional/surgical therapy, and fatal outcome [23]. We also recorded the last documented clinical visit and the eventual date of death within one year after the biopsy date.

### Histopathological data

Histopathological data was collected from the pathological report including a judgment of tissue quantity and the resulting histopathological reliability. If the specimen showed alterations of liver architecture, the dignity (malignant, benign) and presumed histopathological differentiation was recorded. Furthermore, fibrosis staging (usually according to METAVIR system) was obtained [24], if the sample included sufficient tissue.

### Statistical analysis

Analyses were conducted and graphics rendered with MedCalc Software (Version 20.2). Data are presented as mean ± standard deviation or median and interquartile range as appropriate. If not further specified, percentages were calculated with the respective subgroup as basis. Sensitivity and specificity for appropriate clinical decision making were calculated for all patients who underwent biopsy (“intention-to-diagnose”). For multivariable analyses, we conducted stepwise logistic regression (for procedural risk factors) and Cox-regression analyses (for survival-related risk factors) with p > 0.2 as the criterion for dropping values and p < 0.1 for entering them. Results were considered significant for p < 0.05 unless other specified.

## Results

### Case identification and study cohort

**Fig 1:**
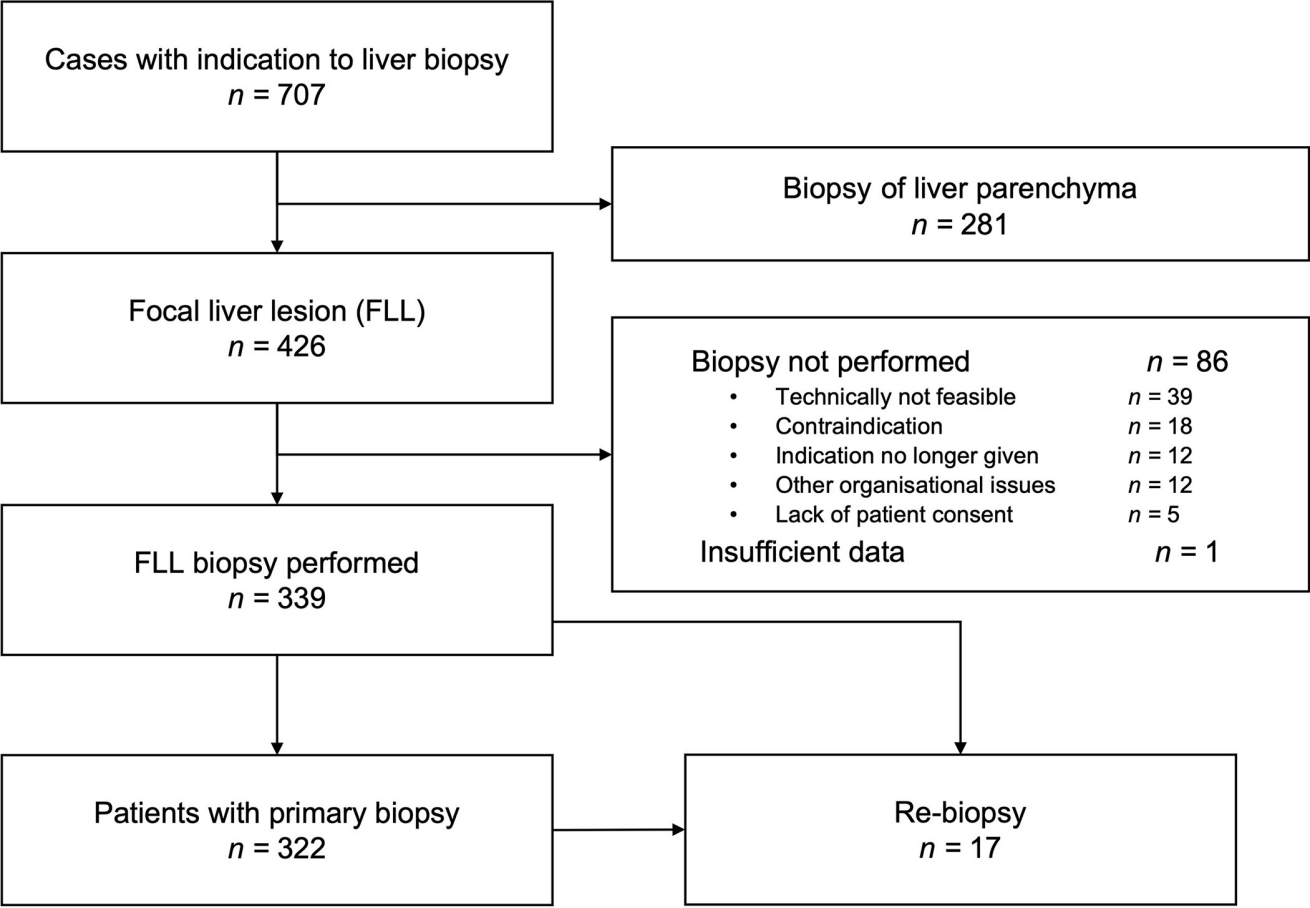
Case identification and selection of study participants.

We identified 426 cases, n = 339 fulfilled the inclusion criteria including 322 primary and 17 repetitive biopsies (Fig 1). The baseline characteristics are shown in Table 1. The clinical indication for the FLL biopsy was a suspected malignant lesion in 309 (96%) cases. Of these, a malignant disease was already established at a distant localization in 157 (49%) cases and the liver biopsy was performed for suspicion of hepatic metastasis.

**Table 1:** Patient characteristics.

Patient characteristics	Primary biopsy *(*n = 322)	Re-biopsy* (n = 17)
Mean age (years; [IQR])	65 [55–73]	73 [57–76]
Female	130 (40.4%)	4 (23.5%)
BMI (kg/m^2^; [IQR]) ^[^^1^^]^	25.4 [22.8–29.4]	26.9 [23.3–29.6]
≤25 / >25≤30 / >30≤35 / >35 (%-distribution)	47.5 / 29.4 / 16.6 / 6.6	47.1 / 29.4 / 11.8 / 11.8
Liver cirrhosis	64 (19.9%)	4 (23.5%)
Child-Pugh class A / B / C (%-distribution)	70.3 / 23.4 / 6.3	100 / 0 / 0
Coronary heart disease	34 (10.6%)	1 (5.9%)
Chronic kidney disease (stage II–V) [KDIGO]	35 (10.9%)	1 (5.9%)
Type 2 diabetes	107 (33.2%)	10 (58.8%)
Established diagnosis of malignant disease	157 (48.8%)	10 (58.8%)
Antiplatelet agents and anticoagulation therapy ^[^^1^^]^		
None	194 (62.8%)	9 (52.9%)
Acetylsalicylic acid		
Continued	23 (7.4%)	1 (5.9%)
Interrupted	28 (9.1%)	1 (5.9%)
P2Y12-Antagonists		
Continued	0	0
Interrupted	9 (2.9%)	0
Therapeutic anticoagulation		
Continued with LMWH	2 (0.6%)	0
Interrupted	90 (29.1%)	7 (41.2%)
Preinterventional substitution of		
Prothrombin complex concentrate (PCC)	7 (2.2%)	0
Platelet transfusion	7 (2.2%)	1 (5.9%)
Other	3 (0.9%)	0
Haemoglobin (mmol/l; [IQR]) ^[^^1^^]^	7.8 [6.7–8.5]	7.6 [6.5–8.5]
Platelets (exp9/l; [IQR]) ^[^^1^^]^	229 [162–302.5]	206 [131–324]
Total bilirubin (μmol/l; [IQR]) ^[^^3^^]^	9.4 [6.6–16.9]	8.7 [6.2–18.3]
Prothrombin time (%; [IQR]) ^[^^1^^]^	88 [76.5–99]	80 [68.5–93]
INR [IQR] ^[^^1^^]^	1.1 [1–1.2]	1.2 [1.1–1.3]
≥ 1.5	13 (4.2%)	0
APTT (s; [IQR]) ^[^^2^^]^	28.4 [26.1–31]	28.5 [27.1–32.8]
Elevated	15 (5.1%)	0
Indication for biopsy		
Probably benign lesion	13 (4%)	0
High suspicion of malignant liver tumour**	173 (53.7%)	10 (58.8%)
Suspected metastasis of previous neoplasm	136 (42.2%)	7 (41.2%)

* Including cases with both primary and repeated re-biopsies

** Including one case with established diagnosis and indication for tumour genotyping

^[1]^ Available in > 95%

^[2]^ Available in > 90%

^[3]^ Available in > 80%

IQR = interquartile range; BMI = body mass index; ASA = acetylsalicylic acid; LMWH = low-molecular-weight heparin; INR = international normalised ratio; APTT = activated partial thromboplastin time

Of the 86 cases with cancelled biopsies, 18 (21%) were not performed due to contraindications: 9/18 (50%) due to ascites, 4/18 (22%) due to poor coagulation and 5/18 (28%) due to other reasons (lack of cooperation or poor general condition).

### Biopsy procedure and characteristics of the target lesion

FLL biopsies were performed using Toshiba Aplio 500 and Canon Aplio i800 devices (Toshiba / Canon Medical Systems, Otawara, Japan) with dedicated convex (PVT 350 BTP) or linear transducers (PLT 308 BTP) with penetrations for needle guidance.

Biopsies were either performed by experienced senior physicians (equivalent to DEGUM level II or higher, n = 6), of whom four performed > 30 FLL biopsies in the study period. Less experienced trainees (n = 4) performed n = 42 biopsies under direct supervision and assistance of an experienced senior physician.

Almost all primary biopsies were performed using 18 G needles (n = 302, 94%). One biopsy pass was performed in 177 (55%) of the patients, 145 (45%) received two or more biopsy passes. In 30 (9%) cases (19 (6%) of them with already established diagnosis of malignant disease), three to five passes were performed.

Lesions were targeted in the right liver in 172 (57%) cases, most frequently in segment V (n = 48, 22%). The majority of patients had multiple liver lesions (n = 215, 68%). Further details of the tissue sampling and lesion selected for biopsy are shown in Table 2.

**Table 2:** Procedural data and characteristics of the target lesion.

Tissue sampling	Primary biopsy *(*n = 322)	Re-biopsy* (n = 17)
Method: Core biopsy needle system ^[321/17]^	314 (97.8%)	16 (94.1%)
16 G / 18 G / 20 G (%-distribution)	2.8 / 94.1 / 0.9	5.9 / 88.2 / 0
Other biopsy instruments ^[321/17]^	7 (2.2%)	1 (5.9%)
Predefined cylindrical length (mm)** ^[261/15]^	49.4 / 41.4 / 9.2	46.7 / 33.3 / 20
33 / 23 / 13 (%-distribution)		
Cases with at least two passes ^[322/17]^	145 (45%)	13 (76.5%)
Location ^[304/16]^	56.6 / 42.1 / 1.3	62.5 / 37.5 / 0
Right / left / both liver (%-distribution)		
Cases performed by an operator with experience in at least 30 other biopsies ^[322/17]^	255 (79.2%)	16 (94.1%)
**Characteristics of the target lesion**		
Solitary lesion ^[318/17]^	103 (32.4%)	3 (17.6%)
Proximity to liver capsule (< 10mm) ^[312/17]^	98 (31.4%)	4 (23.5%)
Proximity to larger blood vessels (< 10mm) ^[314/17]^	32 (10.2%)	0
Median diameter (mm; [IQR]) ^[312/17]^	30 [19–50]	30 [18–45]

* Including cases with both primary and repeated re-biopsies

** Of the core biopsy system

^[x/y]^ Number of available values of primary biopsies/of rebiopsies

G = gauge; IQR = interquartile range

In 55 (17%) cases, periinterventional CEUS was used to guide the biopsy process. The remainder cases underwent biopsy using conventional B-Mode and duplex sonography.

### Histopathology

The quality of the specimen was sufficient for diagnostic decision in the majority of cases. Only in 22 (7%) cases, the sample quantity was not sufficient (n = 17, 5%) or the sample quality was deemed poor (n = 5, 2%). In 245 (82%) cases, the specimen was classified as definitely malignant, including 78 (26%) cases with diagnosis of a primary liver tumour, and 157 (53%) with liver metastasis of extrahepatic disease (Table 3). Rare other malignant neoplasms were lymphomas or gastrointestinal stroma tumours. Benign lesions were mostly haemangiomas, focal nodular hyperplasia, or hepatocellular adenomas. In 32 cases, the histopathological report described normal liver parenchyma without evidence of any focal lesion. In addition to the lesion samples, a definitive fibrosis staging of liver tissue fragments or specimens was available in 185 (70%) cases, and steatosis grading in 190 (71%) cases out of 264 respectively 269 histopathological findings that contained a statement given by the pathologist (Table 3).

**Table 3:** Histopathological results.

Pathological analysis	Primary biopsy (n = 322)	Re-biopsy* (n = 17)
Sample quantity ^[321/17]^	293 (91.3%) / 11 (3.4%) / 17 (5.3%)	14 (82.4%) / 2 (11.8%) / 1 (5.9%)
Sufficient / borderline / insufficient		
Sample quality ^[304/16]^	285 (93.8%) / 14 (4.6%) / 5 (1.6%)	12 (75%) / 3 (18.8%) / 1 (6.3%)
Diagnostic / conditionally diagnostic / not diagnostic		
Histopathological differentiation ^[299/15]^		
Malignant	245 (81.9%)	8 (53.3%)
Hepatocellular carcinoma	49 (16.4%)	2 (13.3%)
Intrahepatic cholangiocarcinoma	29 (9.7%)	1 (6.7%)
Adenocarcinoma metastasis	106 (35.5%)	2 (13.3%)
Melanoma metastasis	10 (3.3%)	0 (0%)
Other metastasis	41 (13.7%)	3 (20%)
Other malignant neoplasm	10 (3.3%)	0 (0%)
Benign liver lesion	15 (5%)	2 (13.3%)
Infectious	7 (2.3%)	0 (0%)
Negative, no focal tissue lesion	32 (10.7%)**	5 (33.3%)
Metastasis: Primary tumour origin ^[157/5]^		
Pancreas	47 (29.9%)	3 (60%)
Pulmonary / bronchial	19 (12.1%)	1 (20%)
Lower gastrointestinal tract	18 (11.5%)	0
Breast	15 (9.6%)	0
Upper gastrointestinal tract	12 (7.6%)	0
Other	33 (21%)	1 (20%)
Staging of liver fibrosis ^[264/15]^		
0–1	121 (45.8%)	8 (53.3%)
2–3	41 (15.5%)	1 (6.7%)
4	23 (8.7%)	3 (20%)
Insufficient sample for staging	79 (29.9%)	3 (20%)
Grading of steatosis ^[269/16]^		
0–1	160 (59.5%)	12 (75%)
2–3	30 (11.2%)	1 (6.3%)
Insufficient sample for grading	79 (29.4%)	3 (18.8%)

* Including cases with both primary and repeated re-biopsies

** 19 (6.4%) of them with not sufficient material amount

^[x/y]^ Number of available values of primary biopsies/of rebiopsies

### Complications

Symptoms during or after the biopsy occurred in 26/322 (8%) of primary biopsy cases, but relevant AEs with extension of the therapy comprised only 5 (2%) minor, 2 (0.6%) major and 1 (0.3%) life-threatening complications.

The most commonly AE was bleeding or liver haematoma (n = 22, 7%). 2 (0.6%) cases received a blood transfusion. The only life-threatening complication was a severe immediate bleeding requiring interventional angiography in a case with a metastasis of a pancreatic neuroendocrine carcinoma. There were no intervention-related deaths.

In all major and life-threatening AEs, morphological evidence of bleeding was detected in the control ultrasound examination. In further 16 (5%) cases, control ultrasound revealed evidence of bleeding (i.e. small hematoma), but without any clinical or therapeutic consequences.

Pain was documented in 7 (2%) cases and 3 (0.9%) had a syncope most likely due to vasovagal reaction. Symptoms after re-biopsies occurred in 2/17 (12%) cases. Interestingly, none of the cases with ≥3 biopsy passes had any recorded AE.

Histopathological characteristics of FLL in cases with occurred symptoms are shown in Table 4.

**Table 4:** Histopathological differentiation of FLL in cases with occurred symptoms.

Histopathological differentiation	Total cohort	Adverse events (n = 26)	Major complications (n = 8)
Malignant	245	22 (9.0%)	6 (2.4%)
Hepatocellular carcinoma	49	5 (10.2%)	2 (4.1%)
Intrahepatic cholangiocarcinoma	29	4 (13.7%)	-
Adenocarcinoma metastasis	106	7 (6.6%)	1 (0.9%)
Melanoma metastasis	10	1 (10%)	-
Other metastasis	41	4 (9.8%)	2 (4.9%)
Other malignant neoplasm	10	1 (10%)	1 (10%)
Haemangioma	4	1 (25%)	1 (25%)
no focal tissue lesion (“negative”)	32	1 (3.1%)	1 (3.1%)
Insufficient material amount	19	2 (10.5%)	-

(Percentages are related to the corresponding cases of the total cohort.)

To analyse risk factors for AEs of primary FLL biopsy, we conducted a stepwise logistic regression analysis including number of passes (1 vs. more), BMI, FLL position (near liver capsule or vessels), suspected entity (liver tumour, metastasis, other) and presence of cirrhosis. The final model only contained “number of passes”, whereby the odds ratio for AEs was 2.32 (95%CI 0.99–5.42, p = 0.052) given ≥2 passes. Severe AEs occurred in 8 cases only. Due to by the low number of AE cases and the monocentric approach, we refrained from further in-depth analyses.

Although the initial study protocol did not consider a systematic collection of data on biopsy tract seeding, no evidence of any tract seeding case was found in the assessed patient files.

### Biopsy success and clinical consequences

**Fig 2:**
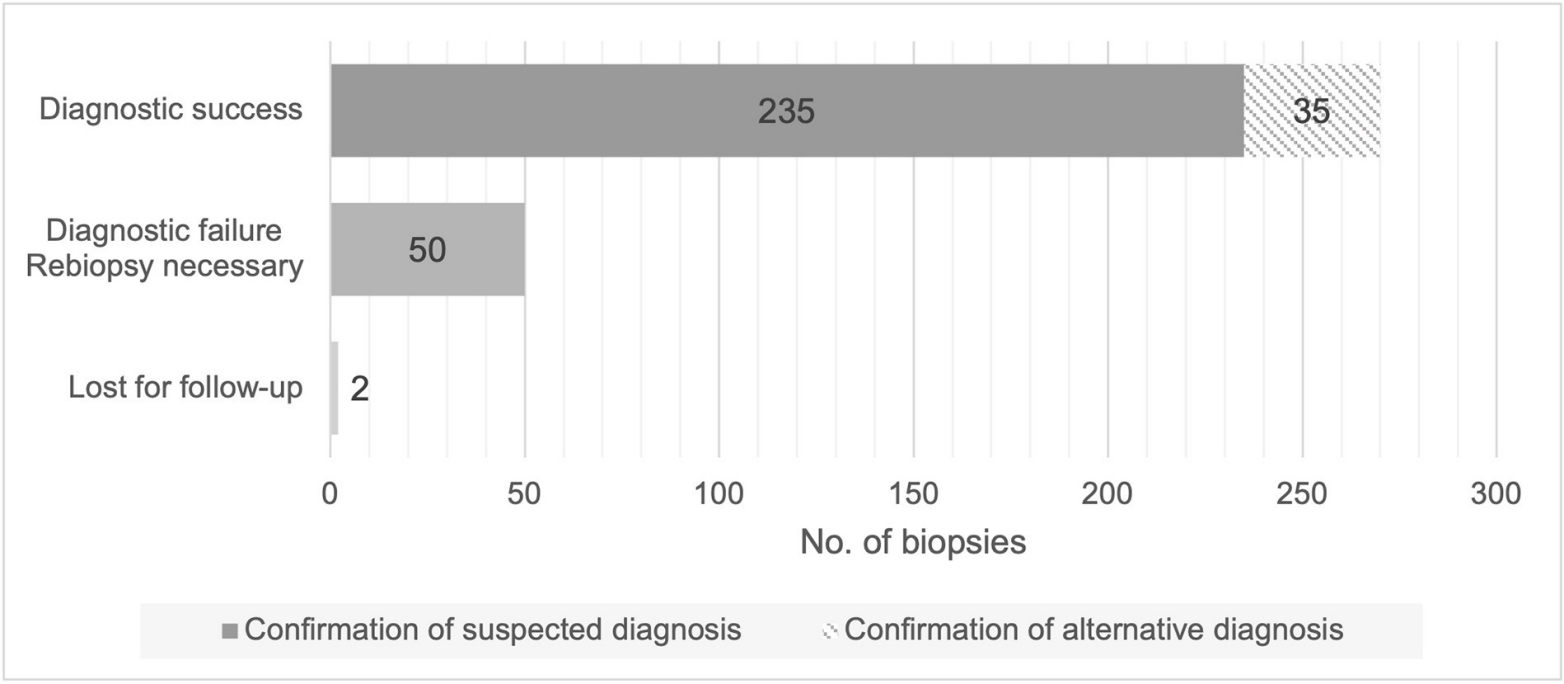
Diagnostic success in primary biopsies (n = 322). No = number.

A sufficient histopathological diagnosis was achieved in 270 (84%) cases, 35 (11%) thereof with an alternative diagnosis than expected (Fig 2).

In 50 (16%) cases, the biopsy did not provide sufficient information for diagnostic decision. In 17 (34%; 5% of the total cohort) of these cases, a second attempt of ultrasound-guided biopsy was performed. These resulted in additional 6 cases with confirmed malignancy. In the subgroup of patients with ≥ 3 passes, 28/30 (93%) provided conclusive diagnostic information, 21 (70%) confirmed the suspected diagnosis. 8/32 (25%) patients with initial histopathological finding of “normal liver tissue” underwent a second biopsy. In 4 (50%) of these cases, a malignant lesion was diagnosed.

To identify factors associated with arriving at a consensus for a clinical diagnosis, we performed a stepwise logistic regression analysis including BMI, lesion size, suspected diagnosis (metastasis, other malignant lesion, other) and examiner experience.

The final model contained only the terms BMI and lesion size. The odds ratio for BMI was 0.60 (95%CI 0.34–1.05, p = 0.074) per 10 BMI points for insufficient diagnosis. The odds ratio for lesion size was 1.12 (95%CI 0.99–1.27, p = 0.081) per 10 mm lesion size.

Periinterventional CEUS use was not associated with biopsy success (42/270 successful vs. 13/50 unsuccessful biopsies, p = 0.085). Given the low case numbers in the respective subgroups, we refrained from including CEUS in the logistic regression.

### Outcome

**Fig 3:**
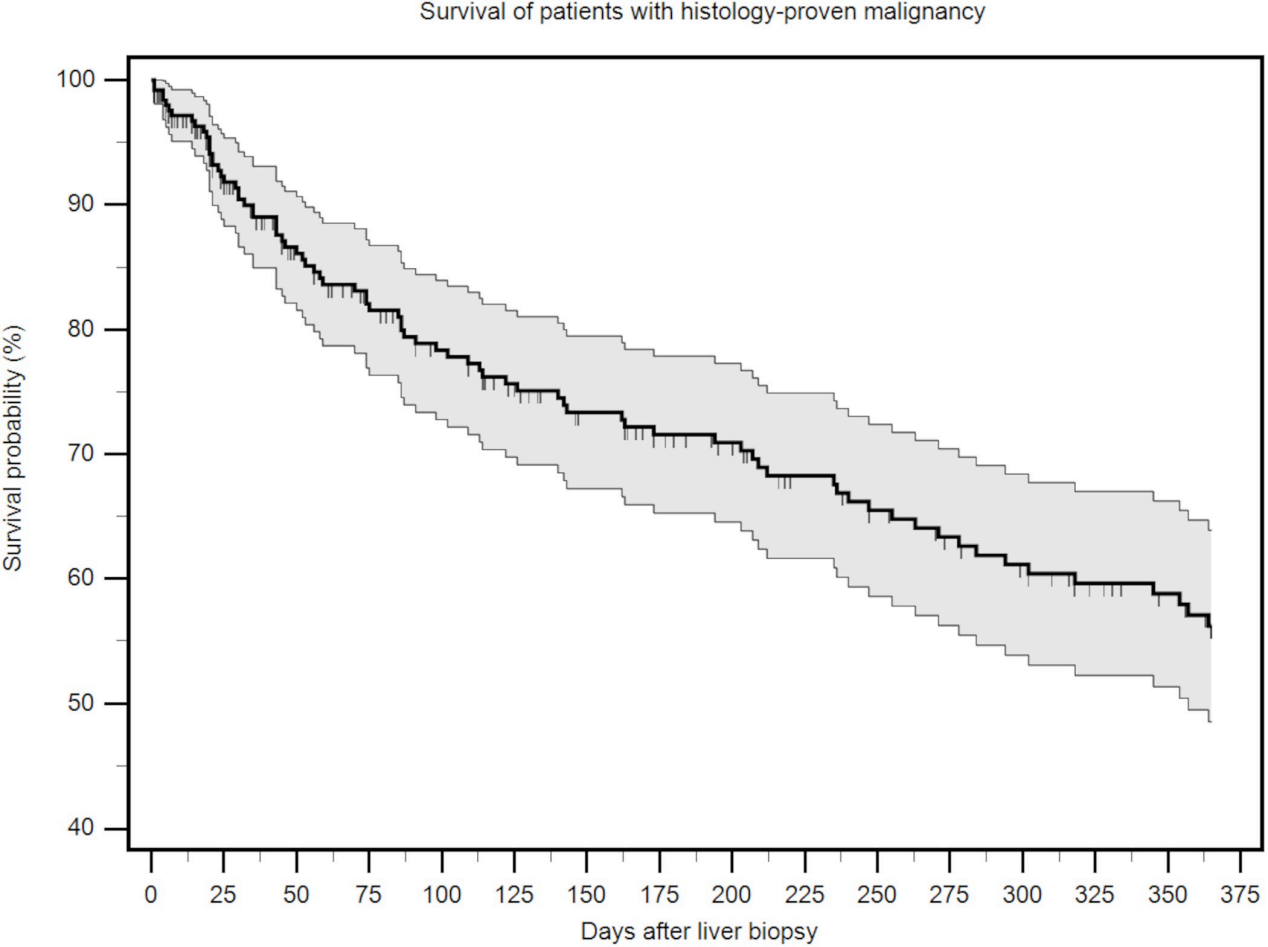
Survival probability of patients with histology-proven malignancy.

Patients were followed-up for a median of 171 (IQR 44–365) days after the diagnostic biopsy. We observed fatal outcome in 93 (29%) cases, of which 81 (87%) died of their primary disease. The remaining patients (n = 12; 13%) died of other not further specified causes not related to the intervention or the underlying disease. Considering only cases with proven malignant lesions, 79 (31%) of the 251 cases died within one year, thereof 44 (18%) within the first 90 days (Fig 3). In the subgroup of cases with ≥ 3 biopsy passes, 8 (27%) patients died, and their median survival was 98 (IQR 38–160) days, which was not significantly different from the remainder cases.

A stepwise Cox-regression analysis including categorical age information and comorbidities for the subgroup of patients with histologically proven diagnosis of a malignant lesion (n = 251) revealed a hazard ratio for all-cause mortality of 1.48 (95%CI 0.95–2.32, p = 0.082) for patients with type 2 diabetes. No other variables were retained in the model.

## Discussion

The continuous development of patient-tailored oncological therapies is largely based on molecular tumour characteristics [25]. The consequential growing need of biopsies of FLL requires a “re-visit” of technical and safety aspects of ultrasound-guided interventions in patient groups dominated by advanced diseases that have not been sufficiently addressed or analysed in previous studies that partially included databases from the first decade of the century (Table 5).

**Table 5:** Comparison of study characteristics and results.

Author	Varela-Ponte R et al. [10]	Mulazzani L et al. [11]	Maheux A et al. [12]	Parente F et al. [13]	Potretzke T et al. [14]	Strobel D et al. [15]	Our study
Year	2022	2021	2019	2018	2018	2015	2023
Country	Spain	Italy	France	Brazil	USA	Germany	Germany
Centres	Single	Single	Single	Single	Single	Multi	Single
No. of focal lesions (% of total case number)	295 (100%)	525 (65.6%)	1283 (53%)	171 (100%)	1107 (59%)	3400 (41.6%)	339 (100%)
Age (years) *median or mean as indicated	69	63	54	62	58	63	65
Females	106 (36%)	296 (37%)	1151 (48%)	82 (48%)	957 (51%)	3576 (43.8%)	192 (59.2%)
BMI [IQR]	-	-	-	-	-	-	25.4 [22.8–29.4]
Cirrhosis	13 (4.3%)	215 (26.9%)	264 (18.2%)	-	-	916 (11.2%)	64 (19.9%)
Type 2 diabetes	-	-	-	-	-	-	107 (33.2%)
Pre-existing malignant disease	132 (44.7%)	419 (52.4%)	-	-	-	-	157 (48.8%)
Minor complication	7 (2.4%)	26 (3.25%)	25 (1%)	4 (2.3%)	13 (0.69%)	443 (5.4%/5903)	5 (1.5%)
Major complication	3 (0.9%)	6 (0.75%)	13 (0.5%)	0	10 (0.5%)	19 (0.6%)	3 (0.9%)
Procedure associated death	0	0	1 (0.04%)	0	0	3 (0.09%)	0
Multiple passes	289 (98%)	108 (13.5%)	381 (29.5%)	-	≥ 3: 116 (34.5%)	2981 (36.5%)	145 (45%)
18 G needle diameter	-	508 (64.1%)	1224 (51%)	171 (100%)	1870 (99.7%)	4185 (51.2%)	302 (94.1%)
Conclusive histology	269 (91.2%)	728 (91%)	1027 (80%)	165 (96.4%)	1033/1107 (93.3%)	-	270 (83.8%)
Malignant histology	214 (72.5%)	-	858 (66.9%)	151 (88.3%)	-	-	245 (81.9%)
Median follow-up after biopsy (days; [IQR])	-	-	-	-	-	-	171 [44–365]

No. = number; BMI = body mass index; IQR = interquartile range; G = gauge

Our data show that the ultrasound-guided approach to FLL histology is a very safe procedure even in elderly patients with a high rate of liver cirrhosis [2]. Our low AE rates are comparable to previous reports from mixed biopsy indications of different organ systems [15] (Table 5). Furthermore, our data confirm the crucial role of FLL biopsies for the diagnostic work-up resulting in a relevant proportion of cases with an unanticipated histopathological result.

The majority of patients had only one biopsy pass reflecting the prudent approach in a cohort with considerable burden of comorbidities. This strategy is confirmed by the finding of a trend towards a higher complication rate in interventions with ≥2 passes, which was the only relevant factor associated with AEs. However, other authors regularly perform multiple passes (Table 5) and achieve comparably safety. This corresponds to our subgroup of patients with three or more passes, were no complication occurred. Careful selection and intervention planning may have contributed to this observation. Therefore, the number of passes remains an individual decision in regard of lesion morphology and patient condition. Also, the operators used almost exclusively 18 G needles which reduce the bleeding risk compared to 16 G needles [2]. Notably, we observed less complications than reported in a recent work from Italy which applied 19G needles in 25% of patients [11]. The case of a life-threatening AE underlines the bleeding risk of non-epithelial malignancies. Although we are not aware of any case on our cohort, the risk of needle tract tumour seeding should be considered before tumour sampling. We applied full core biopsy systems that are correlated with a very low risk of tumour seeding [26, 27].

The observation strategies after FLL biopsy vary in clinical practise. Current recommendations include a monitoring for at least 4–6 hours [28], which may be extended in cases with increased risk of bleeding. Control ultrasound examinations are not generally recommended in asymptomatic cases but have been implemented in this study on the subsequent day prior to patient discharge. This ensured a high probability of having recognised all bleeding complications even in asymptomatic patients.

At our centre, the main indications for FLL biopsy were liver metastases (42%) and malignant primary liver tumours (54%), whereas probably benign liver lesions were approached only in a small proportion of cases. This likely reflects the routine use of optimized non-invasive diagnostic means such as CEUS, which considerably reduces the need for invasive diagnosis [29, 30]. CEUS was also used as periinterventional guidance in selected cases (17%), but case numbers did not allow an in-depth analysis of its impact on diagnostic accuracy of FLL biopsy. For the total cohort, the ratio of unsatisfactory diagnostic results is also considerable (50/320) and potentially related to obesity, which may become more relevant in the future [31]. Of note, in four patients with an initial “normal liver tissue” in the histopathological report malignancy was detected in a 2nd biopsy procedure. This underscores the importance of careful assessment of plausibility of “negative biopsy results”.

The majority of our patients suffered from malignant diseases of liver and the gastrointestinal system which frequently require a complementary endoscopic work-up. This supports the common practice that ultrasound-guided biopsies can be performed by clinical physicians themselves [5].

To the best of our knowledge, this study reports the first structured long-term follow-up data concerning survival after FLL biopsy. Such information is clinically relevant because malignant FLL often define advanced tumour stages with restricted prognosis and potential benefit of the histopathological analysis must be weight out against the interventional risk. A relevant proportion of our patients with a malignant FLL died within one year, but the short-term mortality was much lower. Although many additional factors including best-supporting care decisions interfere with these observations, our data imply that FLL biopsy indication is usually justified even in patients with advanced diseases as well as in cases with necessity of more than two biopsy passes. However, a careful individual decision making remains essential, especially in patients with diabetes mellitus. Complex molecular analyses of advanced malignancies will become more relevant in future therapeutic concepts for cancer patients. This necessitates very large specimens, which were required, however, only in few patients of our cohort. Nevertheless, our data indicate that such interventions may be safe if performed cautiously. Future prospective data on the risk-benefit-ratio of large biopsy specimens are warranted.

A single centre retrospective analysis has inherent limitations. Our institution houses a large comprehensive cancer centre, whereby the rate of biopsy indications in advanced oncological diseases may be higher than in the average routine care. In addition, the ultrasound unit is a certified ultrasound training centre with experienced interventionists [5], which can influence AE rate and threshold definitions of technical limitations for biopsy, e.g. by preferences of needle type choice. The follow-up data show censored cases, which impaired the detailed analysis of factors associated with early mortality and the assessment of false-negative benign biopsy-results. Nevertheless, our study is one of the largest databases dedicated to biopsy of FLL (Table 5). In the future, our findings should be verified in larger multicentric registries, that are already collecting data [32].

In conclusion, ultrasound-guided FLL biopsy is an efficient and safe diagnostic measure, even for patients with advanced diseases. The limitations of the procedure and its associated risks should be considered in patients with advanced malignancies.
